# The Fungus *Metarhizium* sp. BCC 4849 Is an Effective and Safe Mycoinsecticide for the Management of Spider Mites and Other Insect Pests

**DOI:** 10.3390/insects13010042

**Published:** 2021-12-30

**Authors:** Rudsamee Wasuwan, Natnapha Phosrithong, Boonhiang Promdonkoy, Duangjai Sangsrakru, Chutima Sonthirod, Sithichoke Tangphatsornruang, Somsak Likhitrattanapisal, Supawadee Ingsriswang, Chettida Srisuksam, Kewarin Klamchao, Malinee Suksangpanomrung, Thipmanee Hleepongpanich, Sareeya Reungpatthanaphong, Morakot Tanticharoen, Alongkorn Amnuaykanjanasin

**Affiliations:** 1 National Center for Genetic Engineering and Biotechnology, National Science and Technology Development Agency, Pathum Thani 12120, Thailand; rudsamee.was@biotec.or.th (R.W.); natnapha.pho@ncr.nstda.or.th (N.P.); boonhiang@biotec.or.th (B.P.); duangjai.san@nstda.or.th (D.S.); chutima.son@nstda.or.th (C.S.); sithichoke.tan@nstda.or.th (S.T.); somsak.lik@biotec.or.th (S.L.); supawadee@biotec.or.th (S.I.); chettida.sri@biotec.or.th (C.S.); kewarin.kla@biotec.or.th (K.K.); malineec@biotec.or.th (M.S.); thipmanee.hle@ncr.nstda.or.th (T.H.); 2 Thailand Institute of Scientific and Technological Research, Pathum Thani 12120, Thailand; sareeya@tistr.or.th; 3 School of Bioresources and Technology, King Mongkut’s University of Technology Thonburi, Bangkok 10140, Thailand; morakot@nstda.or.th

**Keywords:** acaricide, hemp, hyphal body, insect pest, *Metarhizium*, shelf life, toxicology

## Abstract

**Simple Summary:**

The spider mite is a destructive pest of various crops during warm and dry conditions in tropical countries, including Thailand. The pest is difficult to control despite synthetic acaricide use. In the field, insect populations gradually develop resistance to synthetic pesticides over long-term use. The use of entomopathogenic fungi is more human- and environmentally friendly. We searched and identified a potential fungal isolate from a culture collection, focusing on the genus *Metarhizium*. *Metarhizium* sp. BCC 4849 not only controls the spider mite but also plays a significant role as a natural regulator of other insect pests. Here, we investigated its infection process on the mite, optimized a conidial formulation for extended shelf life, and conducted toxicological assays in animals to assess its biosafety in humans. The fungal genome has been sequenced. The genomic data indicated that oxidoreduction proteins; zinc-, heme-, and iron-binding proteins; and transmembrane transporters are abundant in the genome.

**Abstract:**

Five isolates of *Metarhizium* sp. were evaluated for their pathogenicity against the spider mite (*Tetranychus truncatus* Ehara) (Acari: Tetranychidae) and *Metarhizium* sp. BCC 4849 resulted in the highest mortality (82%) on the 5th day post-inoculation (DPI). Subsequent insect bioassay data indicated similar high virulence against five other insects: African red mites (*Eutetranychus africanus* Tucker) (Acari: Tetranychidae), bean aphid (*Aphis craccivora* Koch) (Hemiptera: Aphididae), cassava mealybug (*Phenacoccus manihoti* Matile-Ferrero) (Hemiptera: Pseudococcidae), sweet potato weevil (*Cylas formicarius* Fabricius) (Coleoptera: Brentidae), and oriental fruit fly (*Bactrocera dorsalis* Hendel) (Diptera: Tephritidae), at mortalities of 92–99%, on 3rd–6th DPI, and in laboratory conditions. The pathogenicity assay against *E. africanus* in hemp plants under greenhouse conditions indicated 85–100% insect mortality on 10th DPI using the fungus alone or in combination with synthetic acaricide. Genome sequencing of *Metarhizium* sp. BCC 4849 revealed the high abundance of proteins associated with zinc-, heme-, and iron-binding; oxidation-reduction; and transmembrane transport, implicating its versatile mode of interaction with the environment and adaptation to various ion homeostasis. The light and scanning electron microscopy indicated that at 24 h post inoculation (PI), adhesion and appressorial formation occurred, notably near the setae. Most infected mites had stopped moving and started dying by 48–72 h PI. Elongated hyphal bodies and oval blastospores were detected in the legs. At 96–120 h PI or longer, dense mycelia and conidial mass had colonized the interior and exterior of dead mites, primarily at the bottom than the upper part. The shelf-life study also indicated that conidial formulation combined with an oxygen-moisture absorber markedly enhanced the viability and germination after storage at 35 °C for four months. The fungus was tested as safe for humans and animals, according to our toxicological assays.

## 1. Introduction

Species in the genus *Metarhizium* are well-known biopesticides for controlling a wide range of insect pests [1]. These pests included phytophagous mites, citrus rust mite, two-spotted spider mite, and bulb mite [2]. The spider mite is an important pest of many crop plants in the greenhouse and the field such as cassava (*Manihot esculenta* Crantz) (Euphorbiales: Euphorbiaceae), durian (*Durio zibethinus* Murray) (Malvales: Bombacaceae), pepper (*Piper nigrum* L.) (Piperales: Piperaceae), tomato (*Solanum lycopersicum* L.) (Solanales: Solanaceae), potato (*Solanum tuberosum* L.) (Solanales: Solanaceae), bean (*Phaseolus vulgaris* L.) (Fabales: Fabaceae), corn (*Zea mays* L.) (Cyperales: Poaceae), strawberry, *Fragaria ananassa* (Weston) Dunchesne (Rosales: Rosaceae), and cannabis (*Canabis indica* L. and *C*. *sativa* L.) (Rosales: Cannabaceae) [3]. Marijuana and hemp had an estimated value of USD 35.8 billion each year and a market value of USD 688 million in 2016, respectively, in the USA [4]. Cassava is also an important economic crop in several tropical countries. Tetranychid mites resulted in devastating losses (14–47% in the field) in cassava in the Philippines, Indonesia, and India [5,6]. Widely-used entomopathogenic fungi belong to species *Hirsutella thompsonii* Fisher (Hypocreales: Ophiocordycipitaceae), *Paecilomyces (Isaria) fumosorosea* Wize (Hypocreales: Cordycipitaceae) [7] *Verticillium (Lecanicillium) lecanii* Zimm (Hypocreales: Cordycipitaceae) [8], *Beauveria bassiana* (Bals.-Criv) Vuill (Hypocreales: Cordycipitaceae), and *Metarhizium robertsii* (Metschn) Sorokin (Hypocreales: Clavicipitaceae) [9,10]. *M. robertsii* was one of the first organisms used for management of agricultural pests. The pioneering immunologist Elie Metchnickoff initiated trials of this fungus against the wheat cockchafer *Anisoplia austriaca* Herbst (Coleoptera: Scarabaeidae) in 1879 [11]. One of the most successful biological control using *Metarhizium* was an application of two million hectares of sugar cane plantations in Brazil with *M. robertsii* to control spittlebugs (*Clastoptera achatina* Germar) (Hemiptera: Clastopteridae) [12].

Infection of *Metarhizium* sp. on insect hosts consists of several stages [13]. The fungal conidia adhere to the waxy cuticle of the host and germinate [14]. The germ tubes can differentiate into the appressorium, from which penetration occurs through the penetration peg. The fungus can degrade and penetrate the insect cuticle using a combination of cuticle-degrading enzymes and mechanical pressure while overcoming any stresses encountered along the way [15,16]. The fungus then colonizes the insect hemolymph, causing the mycosis of insects. When the host dies, the hyphae extrude the cadaver to the outer environment and form a dense mycelial network and green spores [17]. This event starts a new round of infection [18].

In the field, insect populations gradually develop resistance to synthetic pesticides. Farmers then increase the dose and finally discover that the pesticides are no longer effective. The field population of whitefly (*Bemisia tabaci* Gennadius) (Hemiptera: Aleyrodidae) developed resistance against pyrethroids, organophosphates, and neonicotinoids after their prolonged use for over 30 years [19]. Resistance against avermectin and pyridaben was detected in the population of the spider mite *Tetranychus truncates* Ehara (Acari: Tetranychidae) and could be inherited by their progeny [20]. An alternative strategy of chemical control needs to be developed. A more human- and environmentally friendly approach is necessary and will reduce the impacts of chemical accumulation in the environment and health risks associated with pesticide use. Entomopathogenic fungi can play a significant role as natural regulators of the insect and mite populations. They can be applied alone or as a combination with synthetic pesticides/acaricides. The combined use of entomopathogenic fungi with a lower dose of chemical insecticides led to a synergistic additive effect and increased mortality of several insect pests. The lower dosage of the chemicals not only enhanced the efficacy of the entomopathogen but also led to relatively short exposure times on the insects [21,22]. In this study, we screened five *Metarhizium* sp. isolates against the cassava mite, *Tetranychus truncatus* Ehara (Acari: Tetranychidae). *Metarhizium* sp. BCC 4849 had the highest pathogenicity against this mite and five other insect pests. This study documented the infection mechanism under laboratory conditions. In addition, the effect of the fungus alone and in combination with acaricide fenpyroximate against African red mite, in hemp plants (*Canabis sativa* L.) (Rosales: Canabaceae) under greenhouse conditions, was evaluated. Shelf life of conidial powder formulations at different temperatures was performed. Lastly, biosafety assessments in animals for the human safety evaluation, including acute oral toxicity, acute dermal toxicity, acute dermal irritation, acute eye irritation, and acute pulmonary toxicity/pathogenicity, were tested.

## 2. Materials and Methods

### 2.1. Fungal Strains

All the fungal isolates used in this study were obtained from Thailand Bioresource Research Center (TBRC). These included *Metarhizium* sp. BCC 30,455, BCC 4849, TBRC 4908, TBRC 4912, and *M. anisopliae* BCC 16,000. Each strain was grown on potato dextrose agar (PDA; Difco, Detroit, MI, USA) in the dark at 25 °C for 12 days. Conidia were scraped from culture media and transferred into 10 mL of sterile water, then suspended in 0.2% tween-80 (Loba Chemie, Mumbai, India). The conidial density was adjusted to 1 × 10^8^ conidia·mL^−1^ for use as inoculum in insect bioassays.

### 2.2. Insect-Rearing Culture

Cassava mite (*T*. *truncates*) was obtained from the Department of Agricultural Extension, Thailand. The adults were maintained on potted bean plants in a greenhouse and reproduced on surface-sterilized mulberry leaves under laboratory conditions at 25–28 °C, 50–70% relative humidity (RH). Bean aphids (*Aphis craccivora* Koch) (Hemiptera: Aphididae), cassava mealybugs (*Phenacoccus manihoti* Matile-Ferrero) (Hemiptera: Pseudococcidae), and sweet potato weevil (*Cylas formicarius* Fabricius) (Coleoptera: Brentidae) were collected from vegetable, cassava, and sweet potato plantations, respectively, for subsequent bioassays. Oriental fruit flies *(Bactrocera dorsalis* Hendel) (Diptera: Tephritidae) were provided by the Thailand Institute of Nuclear Technology and reared on a sucrose-yeast extract-containing substrate.

### 2.3. Microscopy of the Infection Process

For light microscopy, cassava mites (*n* = 20–30) were inoculated by topical application with 0.2% coconut oil (Bioagricert, Samut Sakhon, Thailand)-mixed conidial suspension of *Metarhizium* sp. BCC 4849 at 1 × 10^8^ conidia·mL^−1^ and placed on mulberry leaves. The treatments were transferred to Petri-dishes and kept in a plastic box at 25 °C, 70–80% RH in darkness. Light microscopy was used to observe the infection process of the *Metarhizium* strain on the insect at 24, 48, 72, 96, and 120 h post-inoculation (PI). Five inoculated mites were observed for distribution and cell types and shape at each time point. The specimens were stained with 2.5 µL lactophenol cotton blue (Sigma-Aldrich, St. Louis, MO, USA) solution (0.5 mg·mL^−1^ methyl blue), mounted with 5 µL 50% glycerol (KemAus, New South Wales, Australia) on a glass slide, and observed under Nikon ECLIPSE Ti2-E (Nikon, New York, NY, USA).

We also performed scanning electron microscopy (SEM) of the inoculated cassava mites as follows. Adult mites were inoculated as described above and fixed with 5% glutaraldehyde (Sigma-Aldrich, St. Louis, MO, USA). The insects were washed three times with phosphate buffer saline (PBS) (Sigma-Aldrich, St. Louis, MO, USA) (pH 6.98) and post-fixed in 2% osmium tetroxide (Electron Microscopy Sciences, Hatfield, PA, USA) in the same buffer for 16 h. Specimens were dehydrated in a gradient series from 30 to 100% ethanol and dried under CO_2_ (critical point dryer model CPD K850; Quorum, East Sussex, United Kingdom). Specimens were mounted on a metal stub, sputter-coated with a layer of gold in a coater model Q150RS (Quorum), and documented under FE-SEM model SU5000 (Hitachi, Tokyo, Japan).

### 2.4. Insect Bioassays

Five isolates in the genus *Metarhizium* were separately applied on adult cassava mites in a single dose of 1 mL of conidial suspension at 1 × 10^8^ conidia·ml^−1^ and maintained on mulberry leaves. For all treatments, including an untreated control, mulberry leaves were placed on sterile tissue papers and moisturized with 2 mL of sterile water in 150 mm Petri dishes. Each treatment had 5 replicates (*n* = 30 for each replicate). The bioassay was repeated twice. Only the *Metarhizium* isolate with the highest virulence against cassava mites was selected for subsequent insect bioassays. 

We performed insect bioassays of *Metarhizium* sp. BCC 4849 (that had the insect virulence) against four other pests, including bean aphid, cassava mealybug, oriental fruit fly, and sweet potato weevil, under laboratory conditions. Bioassays against the third nymphal stages of bean aphid and cassava mealybug (five replicates; *n* = 20 each) were conducted as described above with some modifications. Bean and cassava leaves were used for feeding bean aphids and mealybugs, respectively, during the assays. For the bioassay against sweet potato weevil, the larval stage of potato weevil was inoculated by a 30-s dip in 1 mL of conidial suspension at 1 × 10^8^ conidia·mL^−1^ and then fed with sweet potato pieces. There were three replicates (*n* = 10 each). Control insects were treated with the same solution without fungal conidia. Mortality data were recorded daily for five days. We also performed a bioassay against adult oriental fruit flies (three replicates; *n* = 20 each). Adult flies were sedated at −20 °C for 15 min before fungal inoculation. For each replicate, 20 fruit flies were gently mixed with 1 g of *Metarhizium* sp. BCC 4849-colonized rice grains in a 50-mL tube for 1 min. Control flies were treated similarly with the rice grains without fungal conidia. Treated flies were transferred to plastic cages that had a feeding substrate containing 3.5 g/L yeast extract and 1 g/L sucrose and water and maintained at 25–30 °C, 70–80% relative humidity (RH), and photoperiod of 10:14 h (L:D). Insect mortality was recorded daily. Fly cadavers were placed in a moistened paper to observe mycosis. For the four pests tested, the pathogenicity assays were repeated twice.

### 2.5. Greenhouse Trials and Application Treatment

We conducted greenhouse trials of *Metarhizium* sp. BCC 4849 against African mite, an important pest of hemp. Fifty potted hemp plants, hybrid varieties RPF1, RPF2, RPF3, and RPF4, were naturally infested with African mites. Two trials were conducted at a greenhouse, Thailand Science Park, from 7 to 17 October 2020 and from 11th to 21st February 2021. Mean temperature, RH, and photoperiod in the greenhouse were 29 ± 1 °C, 75%, and 10:14 h (L: D), respectively. Preventing an outbreak of red mites in the greenhouse was a high priority. Consequently, an untreated control was not included in this study. Therefore, we tested two fungal treatments in the greenhouse trials. The fungus *Metarhizium* sp. BCC 4849 was applied alone or in combination with 0.002% (*w*/*v*) acaricide fenpyroximate. The conidial suspension was prepared in 0.025% APSA-80 (Amway, Ada, MI, USA). The fungal treatment was applied three times at a 3-d interval. The *E. africanus* population was determined one day before application and after the 1st, 2nd, and 3rd application. For the insect determination in each treatment, five leaves were randomly picked at the middle and bottom sections of the hemp plant three days after each application and microscopically checked for the number of live and dead nymphs and adults per leaf under a stereo microscope EZ4 HD (Leica, Wetzlar, Germany). All leaves were placed on moist paper in a 150 × 15 mm Petri dish to observe mycosis.

### 2.6. Determination of Conidiation in Different Substrates 

The conidial suspension was prepared by dislodging from 12-d-old culture and adjusted to a final concentration of 1 × 10^7^ conidia·mL^−1^. This was used as an inoculum for the determination of conidiation in different culture media. These media included PDA, half-strength PDA, malt extract agar (MEA), oatmeal agar (OMA), and Sabouraud dextrose agar supplemented with 1% yeast extract (SDAY). All the cultures were conducted in 90 × 15 mm Petri dishes by transferring and spreading 100 µL of the conidial suspension and incubating at 25, 28, and 30 °C. After incubation for 7 and 10 days, conidia were harvested by dislodging as described above, in 10 mL of 0.2% tween-80 (Loba Chemie, Mumbai, India) in sterile water. Conidial densities were determined using a hemocytometer. There were two replicates for each treatment, and the experiment was repeated twice.

### 2.7. Conidial Powder Formulation and Shelf-Life Assay

*Metarhizium* sp. BCC 4849 conidia were grown in rice, the substrate for large-scale production. Aliquots of 200 g of rice grains in polyethylene bags were sterilized in an autoclave for 20 min at 121 °C. The substrate was inoculated with 1 mL of conidial suspension at 1 × 10^8^ conidia·mL^−1^ and incubated at 25–28 °C for 14 days. Conidia were then harvested from 200 g of the rice grains by resuspending in 300 mL of 0.2% tween-80 and precipitation by centrifugation at 3857 g for 10 min. After removal of the supernatant, the conidial pellet was resuspended in 30 mL of 0.2% tween-80, mixed with 100 g of sterile kaolin (Chemipan, Bangkok, Thailand), and air-dried in a biosafety cabinet class II NU440–400E (Nuaire, Plymouth, MN, USA) for 3 days to reduce the moisture content to ~5%, optimal for long-term storage. Aliquots of 50 g of conidia powder formulation were transferred to aluminized pouches. A dual-purpose oxygen/moisture-absorbing sachet (RP-3AN, Mitsubishi Gas Chemical Co., Tokyo, Japan) was enclosed in each pouch and sealed by heat. The conidia were incubated at different temperatures—25 and 35 °C. The control included the fungal conidia prepared as above and kept without the oxygen/moisture-absorbing sachet. There were three replicates for each treatment. The experiment was repeated twice. The conidial survival was performed by the determination of colony-forming units at 2, 4, 8, 10, 12, 14, and 16 weeks.

### 2.8. Toxicological Testing in Animal Models

Acute oral toxicity, acute dermal toxicity, acute dermal irritation/corrosion, and acute eye irritation/corrosion were performed according to Guidelines No. 423, 402, 404, and 405, respectively. All animal models were maintained and tested by Naresuan University Center for Animal Research. In addition, acute pulmonary toxicity/pathogenicity study was performed according to the Microbial Pesticide Test Guidelines of the U.S. EPA (OCSPP) 885.3150. Tier I. (1996) by Jai Research Foundation, India. For acute oral toxicity test, all rats were orally administered with the conidial powder of *Metarhizium* sp. BCC 4849, as described above, at the doses of 300 and 2000 mg/kg body weight. They were then observed for toxicity signs and symptoms for 14 days after administration. For acute dermal toxicity, *Metarhizium* sp. BCC 4849 at doses of 200, 1000, and 2000 mg/kg body weight was applied dermally to the shaved area of the dorsal skin of rats. They were observed for toxicity signs and symptoms for 14 days after patch application. For acute dermal and acute eye irritation/corrosion, *Metarhizium* sp. BCC 4849 was tested on the New Zealand white rabbits to observe the irritation of skin and eye. They were observed for toxicity signs and symptoms, including erythema and edema at 14 and 21 days after patch application into the skin and instillation into the eye. For acute pulmonary toxicity/pathogenicity study of *Metarhizium* sp. BCC 4849, the rats were given a single pulmonary (nasal instillation) dose of fungal conidia in PBS at 1.5 × 10^8^ CFU/rat. Rats were observed for 21 days with interim sacrifices scheduled on days 0, 3, 7, and 14, and at the terminal sacrifice on day 21.

### 2.9. De Novo Genome Assembly of Metarhizium sp. BCC 4849 and Gene Annotation

The genomic DNA of *Metarhizium* sp. BCC 4849 was extracted and used to prepare a SMRTbell library with an insert size of approximately 10,000 bp according to the manufacturer’s instruction (Pacific Biosciences, Menlo Park, CA, USA). DNA sequencing was performed with P6-C4 polymerase and chemistry using 360-min movie times on a PacBio RSII system (Pacific Biosciences). A total of 244,060 raw reads from 4 SMRT cells were corrected, and de novo assembled using the HGAP.3 protocol with default settings from the SMRTanalysis (v2.3) software package (Pacific Bioscience, Menlo Park, California, CA, USA). To obtain the genome draft with the highest quality, genome polishing was performed using Arrow and Quiver from the SMRTanalysis (v2.3.0). The completeness of the final genome assemblies was assessed using Benchmarking Universal Single-Copy Orthologs (BUSCO) (Swiss Institute of Bioinformatics, Lausanne, Switzerland) with the Fungi OrthoDB release 9. The *Metarhizium* sp. BCC 4849 genome annotations were created using the Augustus V3.2.1 program (Bioinformatics Group of the Institute for Mathematics and Computer Science of the University of Greifswald, Greifswald, Germany) using *Metarhizium anisopliae* as a gene model.

### 2.10. Statistical Analyses

All experiments in this study were repeated 2–5 times. There were three replicates in each experiment. Data were analyzed for statistical significance using ANOVA in SPSS package version 11.5 and Student’s *t*-test (IBM, New York, NY, USA).

## 3. Results

### 3.1. Virulence of Metarhizium sp. Strains against T. truncatus and Four Other Pests

Among the five isolates tested, the isolate *Metarhizium* sp. BCC 4849 showed higher insect mortality (82%) than the other isolates (Table 1). Furthermore, the isolate also had similarly high virulence against the nymphs of bean aphid and cassava mealybug; the larvae of sweet potato weevils; and the fruit fly adults at 96, 92, 92, and 99% insect mortality, respectively, on 3th–6th DPI depending on the developmental stages and sizes of target pests (Table 2). The insects in untreated controls had no or very low mortalities during the experimental periods.

### 3.2. Metarhizium sp. BCC 4849 Infection of the Cassava Mites

**Figure 1:**
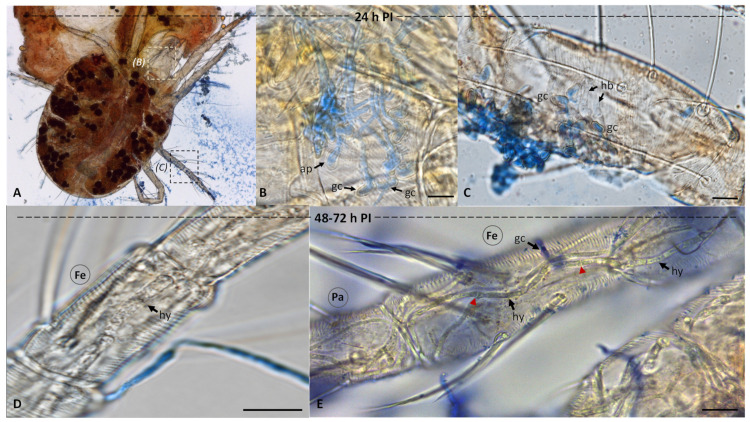
Light micrographs of cassava mites infected with *Metarhizium* sp. BCC 4849 stained with lactophenol blue 24–72 h post-inoculation (PI). (**A**) At 24 PI, a fungal-colonized mite was observed. (**B**,**C**) Germinating conidia (gc), appressoria (ap), and hyphal bodies (hb) were found at the side of the insect body and the leg, respectively. The infection sites were close to the bases of seta on a living mite, Bars, 10 µm. (**D**) At 48–72 h PI, we observed dead cassava mites filled with *Metarhizium* sp. BCC 4849 cells. The hyphae (hy) were found in the hemocoel at the femur (Fe). Bar, 10 µm. (**E**) A network of several hyphae colonized the femur and patella (Pa). Bar, 20 µm.

The lactophenol cotton stained the conidia and fungal cells located outside the insect body blue, which made it easier to differentiate the fungal cells formed inside the insect as unstained, transparent cells. After inoculation by 24 h, all the cassava mites were still alive. Several conidia had germinated, and some produced appressoria on the cuticle (Figure 1A,B). Conidial germination and appressorial formation were also detected at the legs and antenna. Unstained hyphal bodies could be observed inside the leg hemocoel (Figure 1C). These in-vivo-produced fungal cells were formed and developed after the fungus gained entry into the host. These cells could have different lengths and appear irregular in shape. Within the femur, long hyphal cells were formed in the leg hemocoel (Figure 1C). By 48 h PI, most inoculated mites had stopped motile and feeding. Infected mites had started to die by 72 h PI. Long hyphae and hyphal bodies multiplied extensively in the leg hemocoel (Figure 1D). Hyphal fragmentation, as a means of asexual reproduction, was also documented (arrowheads in Figure 1E). 

**Figure 2:**
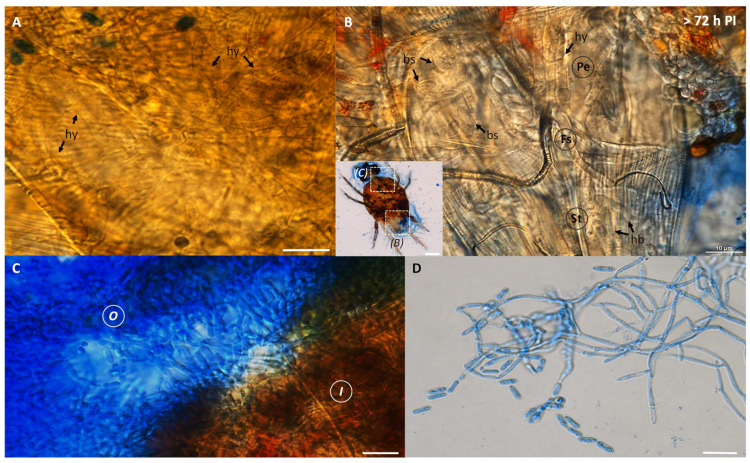
Light micrographs of dead cassava mites at a period longer than 72 h PI. (**A**) Several hyphae (hy) were observed inside the insect body. (**B**) A close-up view of anterodorsal section, including the feeding stylets (Fs), peritreme (Pe), and stylophore (St), showed numerous blastospores (bs) and hyphal bodies. (**C**,**D**) Dense mycelia and a mass of conidia appeared inside (I) and outside (O) of the hosts. Chains of cylindrical-shaped conidia Metarhizium sp. BCC 4849 were also found at the exterior of the dead host. Bars, 10 µm.

The light microscopy also indicated that by 72–96 h after inoculation, internal organs and tissues of infected mites had become heavily colonized by the fungus. An extensive hyphal network was observed inside the infected mites (Figure 2A). Several oval-shaped blastospores, cylindrical-shaped hyphal bodies, and hyphae were present in the anterodorsal (Figure 2B). The extrusion occurred after 120 h PI. Dense mycelia had colonized the interior of dead mites—mycelia emerging from cadavers and forming a mass of conidia (Figure 2C). At the exterior, the fungal conidia could be seen forming in a chain (Figure 2D). After the insect death, macroscopic observation indicated mycelial growth and conidiogenesis on the cadavers, shown by the white and greenish colorations on the body surface, respectively.

**Figure 3:**
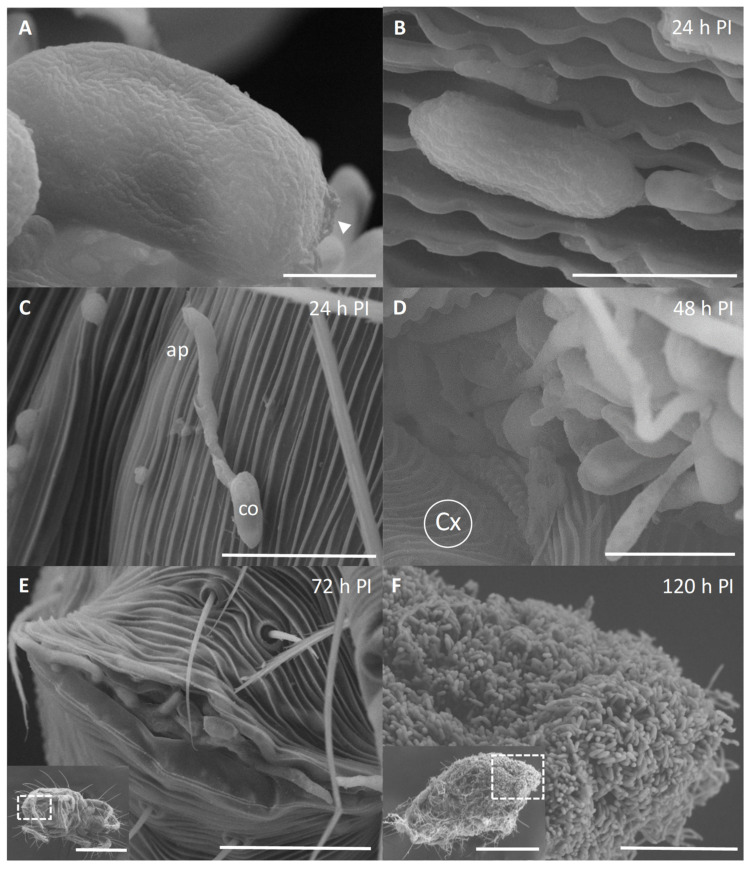
Scanning Electron micrographs of *Metarhizium* sp. BCC 4849 growing on a surface of cassava mites. (**A**) The arrangement of rodlet bundles on the surface of *Metarhizium* sp. BCC 4849 conidia. A conidial scar was seen after abstriction. Bar, 1 µm. (**B**) A germinating conidium was embedded in the striae integumentary lobes of the mite cuticle. Bar, 3 µm. (**C**) At 24 h PI, a germinating conidium (co) formed appressorium (ap) on the cuticle. Bar, 10 µm. (**D**) Conidia and hyphae extensively grew and developed for penetration at the socket of coxa (Cx) at 48 h PI. Bar, 5 µm. (**E**) At 72 h PI, fungal cells and hyphae extruded from the anal opening (indicated by the square in the inset; bar 200 µm). Bar, 20 µm. (**F**) Numerous conidia were present around the insect body, especially opisthosoma (indicated by the square in the inset; bar 200 µm). Bar, 10 µm.

The SEM demonstrated that the conidial surface of *Metarhizium* sp. BCC 4829 had the rodlet bundles packed and arranged tightly throughout the surface (Figure 3A). These fascicle rodlet bundles are a structural feature composed of hydrophobin proteins, which are crucial for adhesion to the host. The microscopic analysis confirmed that the conidia adhered to nearly all the insect body parts. The oblong-to-cylindrical-shaped conidia were also suitable for attachment with the striae and integumentary lobes of the mite surface (Figure 2B). The conidia germinated and developed into the germ tube on the epicuticle (Figure 3B,C) and formed appressorium (Figure 3C) by 24 h PI. At 48 h PI, most conidia germinated, developed into hyphae, and penetrated at the socket between coxa (Figure 3D). The fungus overcame the host immunity and grew extensively inside the mites, as demonstrated above (Figure 1 and Figure 2). After the host died, the fungus extruded the cadavers through openings, such as anal valves (Figure 3E), anal opening, or body cracks, at 72 h PI or longer. By 120 h PI, dense mycelia and conidia had colonized the interior and exterior of cadavers. We observed more clump of conidia at the bottom part of the body than the upper part (Figure 3F).

### 3.3. Efficacy of Metarhizium sp. and Diluted Acaricide Applications on E. africanus Mortality in Greenhouse

**Figure 4:**
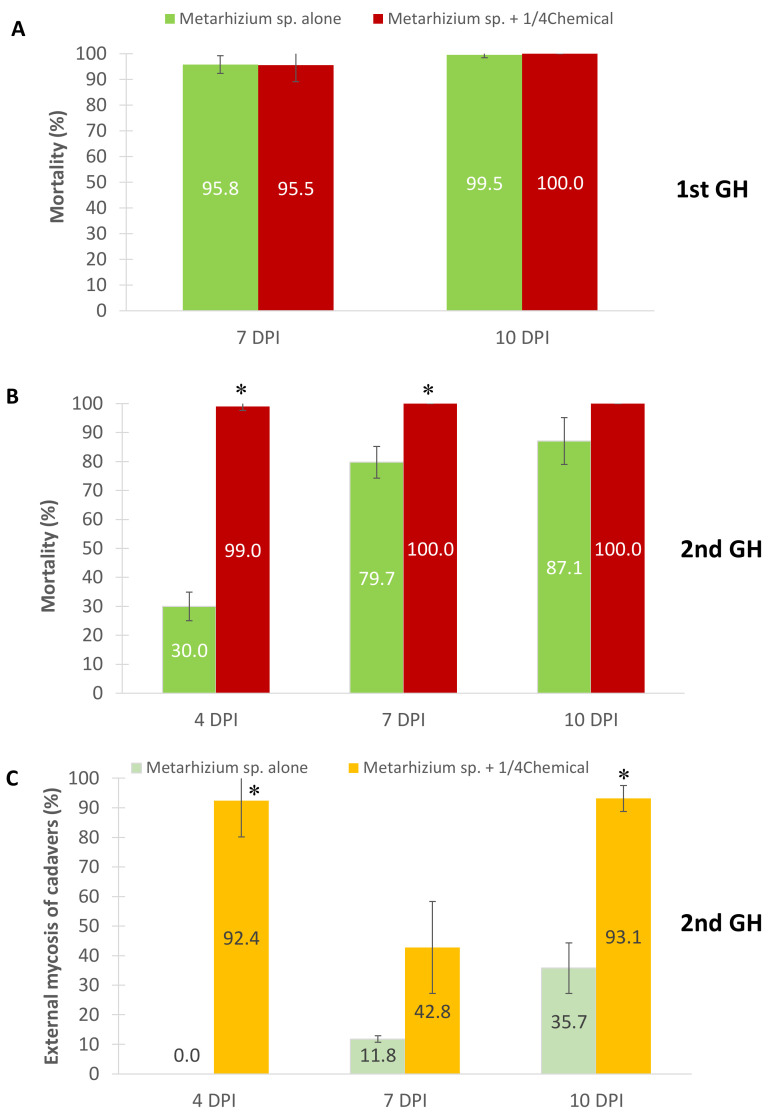
Greenhouse trials of *Metarhizium* sp. BCC 4849 against the African mite E. africanus, an important pest of hemp plants. (**A**,**B**) The first and second trials of the treatment of the fungus alone and the fungus combined with a one-fourth dose of synthetic acaricide, respectively. (**C**) Mycosis percentage of the cadavers in the second trial. Data shown are mean ± s.d. Asterisks indicate statistical significance between two treatments (Student’s *t*-test: *, *p* < 0.05).

The first greenhouse trial of topical application showed similarly high mortality of *E. africanus* in the two treatments. On the 7th day after application, the mortalities of red mites were 95–96% in the fungus-only treatment and the fungus–fenpyroximate combined treatment (Figure 4A). At the end of the observation period on day 10 PI, the mite mortalities were 99–100%, indicating that the fungus killed all the insects regardless of the chemical use at this time point.

In the second trial, high mortalities were also found in the mites treated by the fungus alone or the fungus and chemical combination on days 7 and 10 after application. However, the mortalities of *Metarhizium*-sp.-BCC-4849-inoculated mites slightly decreased to 80–87%. Furthermore, application of the fungus alone caused a marked and significant (*p* = 0.03) decrease of mite mortality on day 4 PI, compared to the fungus-chemical combination (Figure 4B). These data suggested that fenpyroximate helped the fungus infect and kill the mites faster than the fungus alone during the early phase of host infection. We also determined the external mycosis of mite cadavers to verify the completion of the fungal infection cycle. The frequencies of mycosis found in the fungus-fenpyroximate treatment were higher than the treatment of the fungus alone throughout the experimental period (Figure 4C). 

### 3.4. Genome Sequencing and Assembly of Metarhizium sp. BCC 4849

The whole-genome shotgun strategy was performed to sequence and assemble *Metarhizium* sp. BCC 4849 genome from PacBio long-read data. A total of 244,060 raw reads (1.73 Gb) represented 43.25× coverage based on the estimated genome size of 40 Mb. We selected 81,983 of the longest reads (25× coverage) as seed reads for base correction and de novo assembly. After genome polishing, we obtained 26 contigs that covered 37.9 Mb with N_50_ length of 3.51 Mb (L50 = 5 scaffolds). To further evaluate the completeness of genome assembly, genome was checked with the program BUSCO using a fungi-specific database of 290 genes. The Metarhizium sp. BCC 4849 genome recovered 99.7% of the highly conserved orthologs in the fungi dataset, with 99.0% identified as “complete” and 0.7% identified as “partial”. The draft whole-genome sequence of Metarhizium sp. BCC 4849 was deposited at GenBank under the accession number MWYT00000000. An ab initio prediction based on M. anisopliae gene model was used for gene prediction. The genome annotations of Metarhizium sp. BCC 4849 indicated 10,559 protein-coding genes. The average gene size was 1503 bp with 2.8 exons per gene.

**Figure 5:**
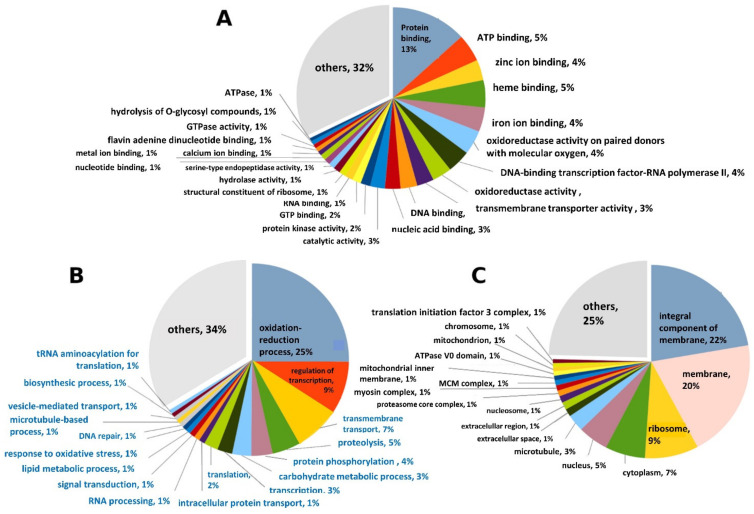
The functional category in the genome of *Metarhizium* sp. BCC 4849 predicted based on the gene ontology as follows: (**A**) molecular function, (**B**) biological process, and (**C**) cellular location.

The functions of 10,559 predicted proteins were annotated using Interproscan version 5.46-81 [23]. The functionally annotated proteins were then categorized based on gene ontology (GO) molecular function, biological process, and cellular location [24] *(*Figure 5). Metarhizium sp. BCC 4849 had approximately 14.5% encoded genes associated with zinc-, heme-, and iron-binding (Figure 5A). Majority (25%) of the proteins were in the oxidation-reduction pathway, which is composed of 120 cytochrome P450 enzymes, 101 short-chain dehydrogenases/reductuases, and 43 zinc-binding dehydrogenases. A relatively high proportion (7%) of the predicted proteins were related to transmembrane transport (Figure 5B). A high proportion of transmembrane transport proteins were in concordance with the predicted cellular locations of proteins. Out of all predicted proteins, the cellular locations of 3580 proteins were annotated. The majority of locations (42.2%) were the cell membrane (Figure 5C). Furthermore, the hidden Markov model scan using SignalP [25] and TMHMM [26] detected 20 proteins with the transmembrane signals. The high abundance of proteins associated with the ion binding and transmembrane transport might implicate a fungal adaptation of ion homeostasis in Metarhizium sp. BCC 4849. 

By comparison to the annotated genome of M. anisopliae ARSEF 549 [27], the Metarhizium sp. BCC 4849 genome had a higher number of genes involved in siroheme biosynthesis, dynein complex, and actin filament depolymerization. In contrast, the genome had fewer genes involved in mercury breakdown processes, such as organomercury catabolism and alkylmercury lyase activity, compared to M. anisopliae ARSEF 549. 

### 3.5. Effect of Subculture Media and Temperature on Sporulation

**Figure 6:**
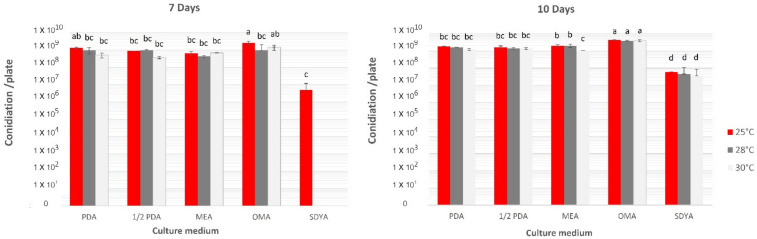
Conidiation of *Metarhizium* sp. BCC 4849 on difference culture media incubated at 25, 28, and 30 °C at days 7 and 10. Data shown are mean ± s.e.m. Different letters indicate statistical significance among the treatments (Tukey’s HSD, *p* < 0.05).

Types of culture media, incubation period, and temperature were determined to optimize conidiation in *Metarhizium* sp. BCC 4849. The fungus grew and conidiated well in nearly all subculture media to 1.7–4.3 × 10^9^ conidia per petri dish except on SDYA after ten days of incubation at 25 °C. The fungal growth on SDYA was mainly the mycelia and few conidia. The oatmeal agar medium generated the highest conidiation, ranging from 3.9–4.3 × 10^9^ conidia per petri dish on the 10th day of incubation, which was significantly different from the other culture media regardless of temperature (25, 28, or 30 °C (Figure 6)). 

### 3.6. Effect of Moisture-Oxygen Absorber Sachets for Shelf-Life Extension

**Figure 7:**
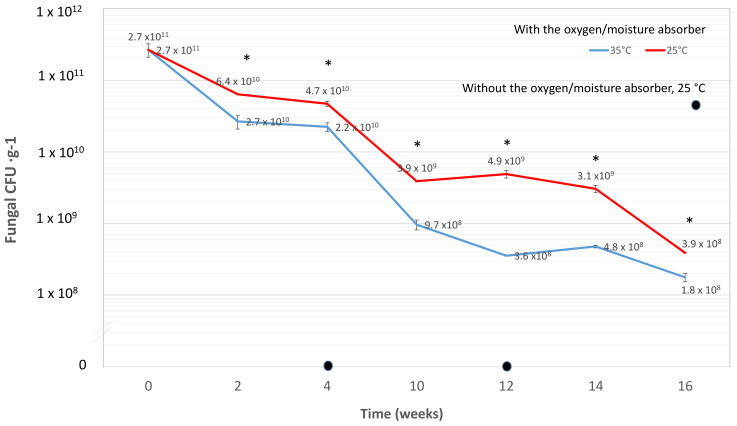
Shelf-life of *Metarhizium* sp. BCC 4849 conidia at two different temperatures of 25 and 35 °C for 16 weeks. Two treatments include the conidia stored with and without the moisture/O_2_ absorber sachet. Data shown are mean ± s.e.m. Asterisks indicate statistical significance between the two treatments (Student’s *t*-test: * *p* < 0.05).

The storage of *Metarhizium* sp. BCC 4849 with the moisture-oxygen absorber RP-3AN tremendously enhanced the shelf-life compared to that without RP-3AN. The fungal CFUs under the storage without RP-3AN were zero at the 4th, 8th, and 12th weeks (Figure 7). In the first eight weeks of storage with RP-3AN, the fungal survival rates at 35 °C were 42–48% of those at 25 °C (Figure 7). Later, the fungal survival rates at 35 °C at the 12th and 14th week decreased to only 7–16% of those at the cooler temperature of 25 °C (Figure 7). These data indicated that the fungus *Metarhizium* sp. BCC 4849 was considerably more viable at the cooler temperature of 25 °C than at 35 °C, the temperature commonly found during the daytime in the tropical region. After storage for 16 weeks at 35 °C, the fungal CFUs were slightly fewer at 1.8 × 10^8^ CFU g^−1^, compared to 3.9 × 10^8^ CFU g^−1^ at 25 °C. The active packaging treatment attributed to prolonged survival of conidia was compared to those without moisture-oxygen absorbers.

### 3.7. Toxicological Testing in Animal Models

For the acute oral toxicity test, the rats did not show abnormal signs of toxicity or mortality after oral administration of *Metarhizium* sp. BCC 4849 at the doses of 300 and 2000 mg/kg body weight. All rats were observed at ½, 1, 2, 3, and 4 h after treatment and once daily for 14 days. Necropsy findings of all rats showed no gross pathological lesions. The oral LD_50_ of *Metarhizium* sp. BCC 4849 in rats was 2000 mg/kg body weight (Table 3). For the acute dermal toxicity test, the rats did not show abnormal signs, symptoms, or mortality after patch application of *Metarhizium* sp. BCC 4849 at doses of 200, 1000, and 2000 mg/kg body weight. After a 14-day observation period, necropsy findings of all rats showed no gross pathological lesions. The dermal LD_50_ of the fungus in rats was 2000 mg/kg body weight. Acute dermal and acute eye irritation/corrosion were observed in New Zealand white rabbits. The results showed that *Metarhizium* sp. BCC 4849 did not cause any irritation of the skin and eye. Moreover, the result of acute pulmonary toxicity/pathogenicity study of *Metarhizium* sp. BCC 4849 showed no mortality and no infectivity and was not pathogenic in rats at a limit dose of 1.05 × 10^8^ CFU/rat. Therefore, the results indicated that *Metarhizium* sp. BCC 4849 did not cause any toxicity in animal models.

## 4. Discussion

**Figure 8:**
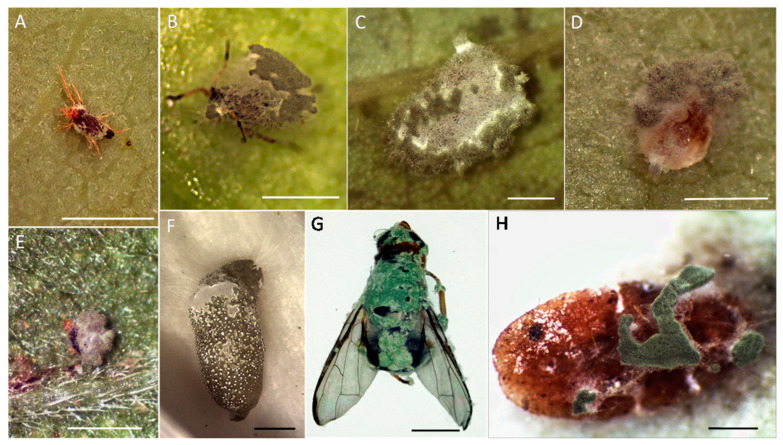
Abundant conidiation and mycelial formation of *Metarhizium* sp. BCC 4849 on various insect cadavers. (**A**) Cassava mite, *Tetranychus truncates*. (**B**) Bean aphid, *Aphis craccivora*. (**C**) Cassava mealybug, *Phenacoccus manihoti*. (**D**) Tobacco whitefly, *Bemisia tabaci*. (**E**) African mite, *Eutetranychys africanus*. (**F**) Sweet potato weevil larvae, *Cylas formicarius*. (**G**) Oriental fruit fly adult, *Bactrocera dorsalis*. (**H**) tapioca scale, *Aonidomytilus albus*. (**A**–**E**) Bars, 0.5 mm. (**F**–**H**) Bars, 2.0 mm.

This study demonstrated that the entomopathogen *Metarhizium* sp. BCC 4849 is a broad-range pathogen capable of infecting several important agricultural pests. These six targeted pests included the bean aphid, cassava mealybug, sweet potato weevil, oriental fruit fly, cassava mite, and African mite, across the orders Hemiptera, Coleoptera, and Diptera, and superorder Acariformes. Furthermore, our preliminary data indicated that the fungus also colonized and killed tapioca scale (*Aonidomytilus albus* Cockerell) (Hemiptera: Diaspididae) and tobacco whitefly (*Bemisia tabaci* Gennadius) (Hemiptera: Aleyrodidae), a critical vector for many plant viral diseases. Mycosed cadavers of these eight targeted insects are shown in Figure 8. 

In the infection of cassava mites, the legs were one of the most frequent sites for fungal colonization. It could be because the legs have a relatively higher density of spines or hair-like structures to anchor conidia than the insect body [28]. For *M*. *robertsii* ARSEF 2575 growing on the surface of *Manduca sexta* caterpillars, the conidia are growing along the cuticle and come across hair sockets, which trigger appressorium production. Hair sockets represent a zone of weakness in the cuticle, which the fungus can exploit [29]. 

For a successful host infection, fungal adhesion and conidial germination occur within 24 h after inoculation. Conidia of several entomopathogenic fungi have the characteristic outer layer. This layer is known as the rodlet later and consists of hydrophobin, the main protein for facilitating the adhesion to the hydrophobic epicuticle [2,30]. The surface properties of aerial conidia from entomopathogenic fungi had the rugose surfaces and were relatively hydrophobic, including those of various strains of *B*. *bassiana* and *M*. *anisopliae* [31], that of *B. bassiana* BCC 2660 [32], and that of *Metarhizium* sp. BCC 4849 in this study. The aerial conidia had varied hydrophobicities that reflected the adaptation of the surface properties to suit their particular habitats and target insects.

Penetration through the cuticle is also vital for fungal infection. The *Metarhizium* enzyme chymoelastase Pr1 has been implicated in cuticle degradation [33]. The authors also speculated *M*. *anisopliae* used up to 78% of protein synthesis to produce this crucial enzyme in the process of penetration. Entomopathogenic fungi also produce chitinases, proteases, and lipases to degrade various layers of the insect cuticle [34]. 

The fungus *Metarhizium* sp. BCC 4849 killed the insects 72 h after inoculation, and the mycelial extrusion happened between 96 and 120 h after inoculation in laboratory, similarly to the previous report of *M*. *anisopliae* [35]. The mycelial extrusion was present initially in the intersegmental areas and later in other areas, resulting in cuticle degradation along the whole body of the insect. Our study showed a large number of mycelia and conidia in the anal cavity and opisthosoma region. Rapid external hyphal development and conidiation of entomopathogenic fungi were observed on two-spotted spider mites under moist conditions [36]. In another study on *B*. *bassiana* and *M*. *anisopliae* infection of subterranean termite *Heterotermes tenuis* using SEM [35], the duration of infection phases ranged between 0 to 144 h PI. The comparative data also showed that penetration, colonization, and conidiogenesis phases were relatively faster for *M*. *anisopliae* than *B*. *bassiana*, leading to higher mortality of *M*. *anisopliae*-inoculated insects.

A limitation in the fungal application for insect/mite control is the lack of a suitable formulation or application strategies for a target host. Combined use of an entomopathogenic fungus with an insecticide/acaricide is an option worth exploring to control the pests. Integrated applications of higher and lower dosages of imidacloprid with *M*. *anisopliae* enhanced the mortality of subterranean burrowers [21]. Application of *M*. *anisopliae* in combination with one µg imidacloprid/g resulted in mortality of 88% of *Cyrtomenus bergi* nymphs compared to 40% for the fungal application alone. In a study of interactions between five synthetic insecticides and five fungal strains, a combination of insecticides with *B*. *bassiana* and *M*. *robertsii* caused higher *Rachiplusia nu* mortalities than any individual agents alone [37]. In our study, we used a combination of an entomopathogenic fungus with a diluted dose of synthetic acaricide. Our result showed that *E*. *africanus* population decreased dramatically after application for 4 days. The mite mortality was nearly 100% in 7 days post application. The lower dose of fenpyroximate enhanced the entomopathogen’s efficacy, leading to the faster mycosis of cadavers.

Alves et al. (2002) [38] proposed a practical requirement for field application is minimal loss of viability after at least three months in storage at 30 °C. However, if a storage of at least twelve months is required, cool storage would be necessary [39]. Among solid and liquid formulation of *M. anisopliae* stored at 30 °C, the solid formulations such as vermicompost, de-oiled castor cake, and farmyard manure formulations retained shelf-life for 210, 190, and 160 days, respectively, compared to gypsum and talc powder that declined by 110 days under storage [40]. In this work, conidia formulated in kaolin-powder formulation and stored with the moisture-oxygen absorber (RP-3A) retained > 10^8^ CFU g^−1^ after four months (120 days) of storage at 35 °C. The revolutionary preservation (RP) system contains a technology capable of effectively absorbing moisture, oxygen, and corrosive gases even under dry conditions [41,42]. The RP is comprised of 5–15% unsaturated organic compound, 10–45% calcium oxide, 10–50% mordenite, 5–15% activated carbon, and 10–30% polyethylene [43]. RP-3A sachets used in this study absorb both moisture and oxygen in a 300-mL air volume. This absorber extends conidia’s shelf life by protecting fungal cells against degradation by oxidation and free water [42]. The effectiveness of RP oxygen and moisture absorbers for extending the shelf life of conidia of *B. bassiana* GHA was demonstrated at a high temperature of 40 °C for six months or at 50 °C for two months [42].

The desired characteristics of a competitive mycopesticide are efficacy comparable to its chemical counterpart, an ability to be used as a component of an IPM program, the stability of the product (long shelf life during transport and storage), low toxicity and ecotoxicity (compared to the chemical pesticide), and simplicity of production and application [44]. Our data indicate that *Metarhizium* sp. BCC 4849 is a promising mycoinsecticide. Its use combined with a lower dose of fenpyroximate is a potential candidate in IPM programs to control pests with pesticides and acaricides. Our findings on the infection processes add to the existing knowledge of how the fungus infects insect hosts. Nonetheless, this IPM strategy should be determined at a broad field level to verify its efficacy.

## Figures and Tables

**Table 1:** Pathogenicity of *Metarhizium* isolates against the spider mite (*Tetranychus truncatus*) at the 5th day post inoculation (dpi).

Isolate	Insect Mortality (%) ± s.e.m.
BCC 4849	82.4 ± 0.1 ^a^
BCC 16,000	7.9 ± 5.4 ^c^
BCC 30,455	32.8 ± 18.1 ^b,c^
TBRC 4912	37.3 ± 16.4 ^b,c^
TBRC 4908	44.9 ± 20.4 ^b^
Control (non-treated)	11.2 ± 8.6 ^b,c^

The results were from two independent experiments. Data shown are mean ± s.e.m. Different letters indicate statistical significance (Tukey’s HSD, *p* < 0.05).

**Table 2:** Virulence of Metarhizium sp. BCC 4849 against various insect pests, inoculation techniques, and effects on insect mortality on days post inoculation (DPI). Conidial suspension was applied at 1 × 10^8^ conidia·mL^−1^.

Target Pest	Inoculation Technique	DPI	Insect Mortality (%) ± s.e.m.
Cassava mite (*Tetranychus truncatus*)	Topical application	5	82.4 ± 0.1
Bean aphid (*Aphis craccivora*)	Topical application	3	96.0 ± 5.5
Cassava mealybug (*Phenacoccus manihoti*)	Topical application	3	92.0 ± 5.7
Sweet potato weevil (*Cylas formicarius*)	Dipping	5	91.9 ± 7.3
Oriental fruit fly (*Bactrocera dorsalis*)	Mixing with dry conidia	6	98.9 ± 1.9

**Table 3:** Biosafety assessment of Metarhizium sp. BCC 4849 conidia in animals.

Study Type	Study Guideline	Animal	Dose Range/Level	GHS ^3^ Toxic Classification
Acute oral toxicity	OECD GLP ^1^ 423	Rats	LD_50_ > 2000 mg/kg	Class 5 (Unclassified)
Acute dermal toxicity	OECD GLP 402	Rats	LD_50_ > 2000 mg/kg	Class 5 (Unclassified)
Acute dermal irritation/Corrosion	OECD GLP 404	Rabbits	-	Class 5 (Unclassified)
Acute eye irritation/Corrosion	OECD GLP 405	Rabbits	-	Category 2B (mild irritant)
Acute pulmonary toxicity/Pathogenecity	U.S. EPA OCSPP ^2^ 885.3150	Rats	No mortality at 1.05 × 10^8^ CFU	

^1^ OECD GLP; Organization for Economic Co-operation and Development: Good Laboratory Practice. ^2^ U.S. EPA OCSPP; United States Environmental Protection Agency recommended for Microbial Pesticide Test Guidelines. ^3^ GHS; Globally Harmonized System of Classification and Labelling of Chemicals (United Nationals, 2017).

## Data Availability

The data presented in this study are available in the manuscript.
